# Design and Field Experimentation of a Cooperative ITS Architecture Based on Distributed RSUs †

**DOI:** 10.3390/s16071147

**Published:** 2016-07-22

**Authors:** Asier Moreno, Eneko Osaba, Enrique Onieva, Asier Perallos, Giovanni Iovino, Pablo Fernández

**Affiliations:** 1 DeustoTech – Fundación Deusto, Avda. Universidades, 24, 48007 Bilbao, Spain; 2 Faculty of Engineering, University of Deusto, Avda. Universidades, 24, 48007, Bilbao, Spain; asier.moreno@deusto.es (A.M.); e.osaba@deusto.es (E.O.); enrique.onieva@deusto.es (E.O.); pablo.fernandez@deusto.es (P.F.); 3 INTECS SpA, Via Umberto Forti 5, 56121 Pisa, Italy; giovanni.iovino@intecs.it

**Keywords:** Intelligent Transportation Systems, Wireless Sensor Networks, distributed intelligence, wireless communications, UbiQuitous computing

## Abstract

This paper describes a new cooperative Intelligent Transportation System architecture that aims to enable collaborative sensing services. The main goal of this architecture is to improve transportation efficiency and performance. The system, which has been proven within the participation in the ICSI (Intelligent Cooperative Sensing for Improved traffic efficiency) European project, encompasses the entire process of capture and management of available road data. For this purpose, it applies a combination of cooperative services and methods for data sensing, acquisition, processing and communication amongst road users, vehicles, infrastructures and related stakeholders. Additionally, the advantages of using the proposed system are exposed. The most important of these advantages is the use of a distributed architecture, moving the system intelligence from the control centre to the peripheral devices. The global architecture of the system is presented, as well as the software design and the interaction between its main components. Finally, functional and operational results observed through the experimentation are described. This experimentation has been carried out in two real scenarios, in Lisbon (Portugal) and Pisa (Italy).

## 1. Introduction

The aim of this work is to define an innovative and fully distributed architecture to enable cooperative sensing in Intelligent Transportation Systems (ITS) environments. The feasibility of the proposed approach has been proven within the participation in the ICSI European project [1], developing a reference end-to-end implementation targeted to both urban and highway scenarios.

The system encompasses the entire process of capture and management of available road data, enabling the generation of services to promote transportation efficiency. The main idea behind the project relies on a local distributed intelligence, operating on a limited geographical scale, where data are timely distributed and processed without the need to contact a central subsystem. For this purpose, the concept of “gateway” (GW) is introduced. Briefly described, a GW is a logical entity that offers capabilities similar to those provided by centralized approaches (e.g., data storage or event processing). The main advantage of the GWs is that they operate on a local scope with the possibility of exchanging messages with other GWs.

This document provides a description of the overall system, defining the high level architecture and the role of its different elements. The way the intelligence and the sensed-data are provided and the concepts behind the design choices are explained.

Moreover, a set of test services developed to validate the system and based on real use cases are described. Besides that, the results of the field trials are shown. These trials have been performed in the cities of Lisbon (Portugal) and Pisa (Italy), with the aim of testing the benefits of the architecture.

In classical transport systems, open loop control governs the interaction between the user/driver and the control centre. In recent years, these systems have been evolving towards ITS [2], where a closed loop interaction between users/drivers and the transportation infrastructure can be found, enabled by cooperative V2X communications and cellular networks [3]. While some of the enabling technologies are entering their mature phase, there is still the need of a complete integrated solution that can take the most benefits from a real-time analysis of the data gathered, and an appropriate reaction on the transportation system.

Nowadays, a centralized architecture for traffic management systems is commonly used. In this case, all the information is gathered by a Traffic Management Centre (TMC) from motorways authorities, weather stations, regional data collection and measurement points on the road. Then, the information is analysed, and decisions are taken in the TMC, sending back to the on-road infrastructure signals. Thus, the architecture of classical ITS systems is purely hierarchical, with sensed data flowing from the leaves (i.e., road-side or vehicle-installed sensors) to the root (i.e., the traffic management centre).

Usually, this kind of approach presents some disadvantages [4]:

- It does not scale adequately with the inclusion of a significant number of new elements and the increase in the amount of data collected, showing a lack of flexibility and making difficult its maintenance.
- This vertical architecture is not suitable to accept and integrate changes in ITS, like new components produced in research centres and in the industry.
- It exhibits latency and security issues related to the centralization of the communications. This latency produces a bottleneck in the system.

For these reasons, research activities in ITS, and especially in C-ITS (Cooperative ITS), have changed the vision behind the definition of new ITS architectures [5], switching from the hierarchical and vertical approach to a new vision, which is more horizontal and distributed [6].

In recent years, important research projects, like CVIS [7], adopted and developed this new strategy to achieve cooperativeness in ITS: vehicles, road-side infra-structure, central systems and personal devices are no longer linked to each other through a static and vertical relationship. Instead, they are seen as “nodes” belonging to a common network. Similar projects like Safespot [8] and COMeSafety^2^ [9] use this approach to increase road safety allowing direct communication between vehicles (C2C). The DRIVE C2X European project [10] is another example of recent implementations of C-ITS architectures, establishing a common reference system for C2X communications and performing successful large-scale field trials. With these new approaches, traffic and travel models and ITS applications specialized to operate on a local scale can be configured in order to fit the needs of the surrounding areas. In addition, they can cooperate exploiting the distribution platform.

The main contribution of the work presented in this paper is not only the design of a new C-ITS architecture, but its practical application and the use of distributed data to make collaborative decisions based on learning capabilities. A set of test services developed to validate the system and based on real use cases are described and used to generate two different real world scenarios. The test results regarding the execution of the use cases in the trial scenarios will be thoroughly described in order to understand the viability of the system.

The remainder of this paper is structured as follows. In the following section (Section 2), the proposed architecture is presented. In Section 3, the main components of the system are deeply described. In Section 4, a description of the test applications and services is made. Additionally, the experimentation carried out is described in Section 5. Finally, conclusions and future work are explained in Section 6.

## 2. Cooperative Decision-Making ITS Architecture

A new cooperative architecture is proposed in this paper. In this architecture, the intelligence of the system is distributed among some of the elements in the infrastructure, which host a software platform for running ITS applications. In line with this, communication with the remote centres happens only for the transmission of aggregated data for long-term operations (e.g., data mining, software upgrades, and logging). On the other hand, real-time data will be processed and stored locally in the system infrastructure, nearby the source of the events.

The developed architecture relies on a local distributed storage and intelligence, which operates on a limited geographical scale without the need to contact the control centres. Moreover, sensed data are processed in a cooperative manner performing content aggregation and integration since the earliest stages. On the other hand, only statistical data for long-term evaluation are sent to the TMC. Two key concepts are defined in order to achieve distributed intelligence and cooperative sensing: the GW and the local/global areas:

- **Gateway (GW).** A GW refers to a physical entity, usually installed inside a Road Side Unit (RSU), which implements the reference architecture, the subsystems Connectors, the Data Distribution Platform (DDP) and the Collaborative Learning Unit (CLU). The functionality of these components will be detailed in Section 3.1, Section 3.2 and Section 3.3, respectively. A GW is able to join Local/Global Areas, and it is connected to different subsystems (VS: Vehicular Subsystems, PS: Personal Subsystems, RS: Road-side Subsystems, etc.)
- **Local/Global Area.** An area is composed of a set of GWs (at least one), communications among these GWs (when more than one is present), and some criteria to define the area perimeter (e.g., based on the density of population, traffic, ICT elements, etc.).

**Figure 1:**
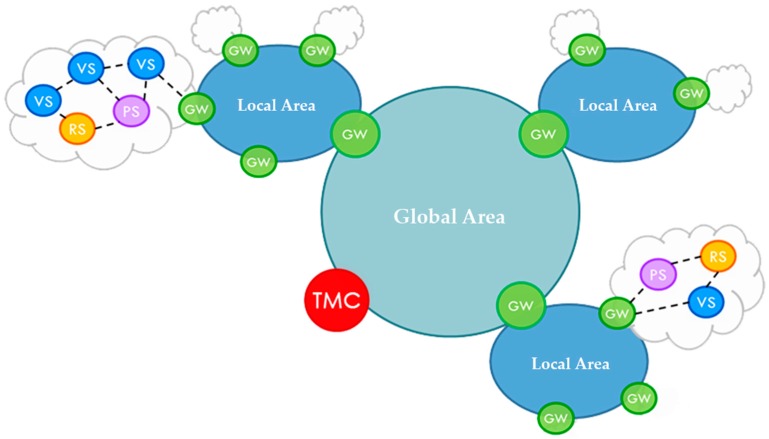
Global system architecture.

A logical view of the architecture, which shows the previously introduced concepts of GW and local/global area, is reported in Figure 1. All of the Local Areas are logically connected to a global one supporting inter-area communication, while physical communication happens through the backhaul technologies and the Internet.

Local storage is offered by each GW of the Local Areas, allowing decisions based also on previous historical data. However, since the applications for real-time processing are mostly concerned about the recent past, only recently sensed data will be kept in the GWs. On the other hand, long-term measurements and storage based on aggregated data for management purposes will be done by the TMC only.

## 3. Software Architecture of Platform on GWs

**Figure 2:**
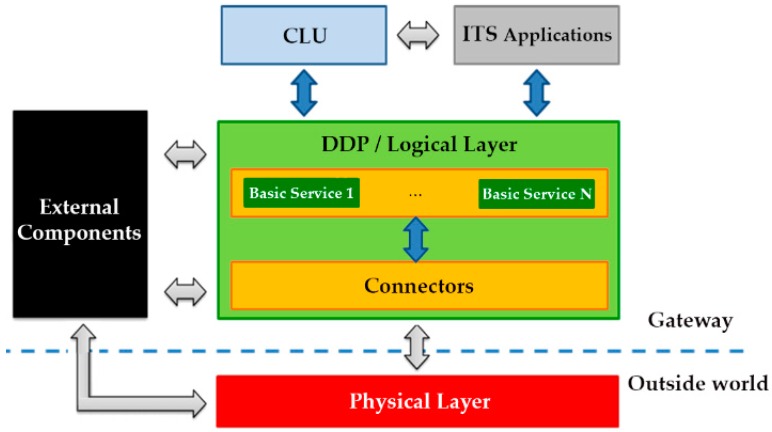
Software Architecture of platform on GWs.

Based on the generated distributed system, the GWs will analyse the gathered information. All this analysis is made autonomously and without requiring online TMC dependences or human participation. They also determine the best traffic strategies for dealing with roadway incidents. In this way, the distributed system enhances the scalability and overcomes the weakness of centralized approaches. For this purpose, each of the GWs hosts a software platform designed for running ITS applications. This software architecture (Figure 2) is composed of various modules classified in logical layers:

- **Connectors.** They are used to extend the GW interoperability via integration interfaces with external subsystems and technologies such as Wireless Sensor Networks, Vehicular Networks, Road-side subsystems, etc. The functionality of the Connectors and the communication between the subsystems and the proposed architecture will be further described in Section 3.1.
- **Data Distribution Platform (DDP)/Logical Layer.** The Logical Layer provides the basic capabilities needed by the ITS services and the CLU in order to interact with the physical layer and the data available. The key component of the logical layer, the DDP, includes the mechanism to communicate between applications and services by managing an events-based architecture. The functionality of the DDP will be further described in Section 3.2.
- **Collaborative Learning Unit (CLU).** Along with the DDP, CLUs are other key software elements of the proposed architecture, which are responsible for providing intelligence to the whole system. These units are able to learn from each situation in order to provide different actions plans in real time. The functionality of the CLUs will be further described in Section 3.3.
- **ITS Services and Applications.** Higher level services or end-user applications implementing traffic and travel models to respond to roadway incidents. An example of the services that can be provided will be shown in Section 4.
- **External Components.** Software components running in the same OS, and are required by other components in order to work properly.

Regarding the runtime environment, the architecture will be executed on a GNU/Linux operating system. The choice of GNU/Linux has been made due to its open-source nature, and due to the success of this OS, which is flexible enough to run on a wide set of hardware.

All of the services, connectors and applications will be provided as OSGi [11] bundles exposing their functionalities to other bundles through the OSGi service layer. Thus, an OSGi reference framework and a JAVA Runtime Environment are required in order to be able to run the GWs software platform.

### 3.1. Connectors and External Subsystems

The GW’s Connectors are responsible for the effective integration of external subsystems into the proposed platform. Each external subsystem providing or receiving data from the GWs needs to be integrated through the development of a specific connector bounded on one side to the DDP, while on the other side providing APIs towards the subsystem components. Thus, each Connector is internally composed of four modules, which are in charge of the following: communication towards the DDP, communication towards the subsystem, configuration, and data model mapping between the subsystem and the DDP.

To enable the above-described mapping functionalities, the Connectors need to implement standard APIs provided by DDP basic services, mainly devoted at enabling a bidirectional communication towards the subsystem behind the connector. Such APIs can be used, for instance, to configure the subsystem components, or to provide feedbacks to devices belonging to the subsystem. They can also use external components that are executed on the same host. External components can be applications, services, bundles or other software elements which have not been designed within the architecture but are required by one or more ITS services in order to properly operate.

The use of Connectors in the GW’s architecture is a powerful solution able to adapt heterogeneous subsystems into the DDP data representation and communications model. Moreover, the use of properly defined Connectors allows the integration of legacy ITS systems in the platform. This fact guaranties the reusability of already developed technologies.

### 3.2. DDP: Data Distribution Platform

The role of the DDP is to enable a scalable and highly adaptable system able to receive data from the architecture subsystems, and to provide a set of capabilities (basic services) used by connectors, ITS applications and other high level services (e.g., the logic behind the CLU).

The DDP has been mainly designed as a set of components capable of interacting among them. Each component can provide services through defined APIs or can use services provided by other components. ITS applications can serve both as applications or high level services extending the capabilities of the DDP’s basic services. Another use of ITS applications is to display processed information to the end-user. These applications exploit the capabilities provided by the DDP’s basic services and can directly communicate with the connectors if a direct interaction with a particular subsystem is needed.

The GW’s reference architecture presented in Figure 2 shows how different architectural components of the DDP interact. The darker interfaces (arrows) in the figure will be defined and implemented by the proposed platform. These will be used by the higher level applications/services and the connectors in order to communicate with the logical layer facilities. The high level interfaces, coloured in light grey, are instead already existing interfaces defined outside the project realm necessary to integrate particular functionalities required by the DDP. Examples of this kind of interfaces are: interfaces required to access external components, interfaces provided by higher level ITS services or applications, and interfaces required to communicate with the external world. The specific architecture connectors use both internal and external interfaces in order to make a bridge between already developed ITS systems and the architecture, as well as among different GW hosts.

The DDP is, therefore, responsible of providing the mechanisms for the communication between the different layers using a publisher/subscriber (P/S) events-based architecture. In this way, other software components would only have to be concerned to inform the DDP about the events in which they are interested. Instead of implementing a high number of point-to-point links between sensors, actuators and GWs at all layers, the DDP will implement a kind of data space, using an appropriate middleware [12], directly supporting many-to-many communications.

The facilities provided by the DDP combined with the logic of services and applications will enable a proper elaboration of all the available information. This fact makes possible the realization of cooperative sensing and ITS, as it is presented in Section 5.

### 3.3. CLU: Collaborative Learning Unit

**Figure 3:**
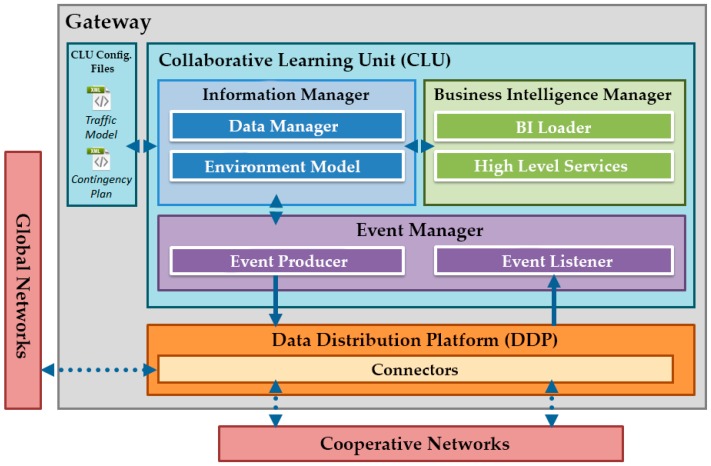
Architecture and technical design of the CLU.

As stated previously, the CLU, as a high level ITS service, is responsible for providing intelligence to the system, executing action plans in real time according to the events received and the current state of the infrastructure. To achieve this autonomous operation, CLUs will include a set of components that will respond to the events received via the DDP (Figure 3).

The distributed traffic management will be achieved through the collaboration of the CLUs in the area enabling cooperative intelligence. The output will also feed up the cooperative networks, resulting in notifications that influence the reality of interest.

Additionally, the CLU itself is composed of three main elements:

- **Events manager**: this module controls the flow of the events at the CLU side; communicating with the DDP to obtain relevant events for the CLU considering its geographical position, the GW’s attached wireless sensor network (WSN), the position of other GWs, etc.
- **Information manager**: this module provides updated information about the GW environment (related sensors, road conditions, etc.) to the CLU services. It also processes the traffic models and the contingency plans configuration files. These two files, updated periodically by the TMC, allow the behaviour change of the CLUs.
- **Business intelligence manager**: this component has a set of high-level services responsible for responding to incidents that arise on the road. These incidents can be, among others, accidents, delays or high CO_2_ levels. In line with this, services implemented by the CLU will have different behaviours, because of the variety of the situations. These behaviours will be dynamically adjusted based on the learning capabilities provided by the artificial intelligence integrated in the CLUs.

#### 3.3.1. CLU Data Model

CLUs will be always updated with new traffic models and contingency plans reported by the TMC. Moreover, using information reported by the system, a decision support system in the TMC will help the traffic manager to update and improve traffic models and contingency plans.

The traffic models consist of two data sets, which may be updated allowing the change in the CLUs behaviour. On the one hand, topological information (position) of the elements within the Local Area of the CLU, as well as the WSN assigned to the same GW, is established. On the other hand, it should also store a table with the set of rules that clearly specify what information is relevant to that particular CLU. In this way, it can properly subscribe to events provided from the DDP. These rule tables may be updated in real time so that the behaviour of the CLU can be adjusted dynamically.

#### 3.3.2. CLU Intelligence

One of the main challenges of this architecture is to develop stable and distributed algorithms based on probabilistic reasoning, and not requiring very high computational resources. As the situations that may occur are rich and varied, services implemented by the CLU will have different behaviours. These behaviours are dynamically adjusted based on the learning capabilities provided by artificial intelligence integrated in the CLUs. In this way, CLUs have to implement some techniques to solve the different problems that could appear. These problems and techniques have been categorized in Table 1 according with the role of the CLU and the kind of problem.

In the category involving routing and regulation cases, any problematic that requires the CLU to take an action or decision is included. With respect to the problems requiring routing decisions, the CLU has to solve routing problems as a necessary task for the implementation of several use cases (presented in Section 4.1). This is because of the fact that almost in every incident detected by the CLU it has to provide a warning signal and (when possible) an alternative route to avoid the inconvenience to the rest of vehicles. The routing problems can have three different natures:

1. **Alternative routes**: this problematic is referred to the classical routing done to face demands received by the CLU. In this category routes given to regular vehicles can be found.
2. **Alternative transport**: these routes involve the parking of the vehicle and the taking of an alternative (public) transportation.
3. **Route guidance and emergency support**: routing made for special vehicles are included here. In this case, special vehicles (emergency and authorities) that must be routed with care have to be considered. It is important to note that these vehicles are allowed to enter any restricted area.

The prediction problems contain all problems and techniques that estimate the probability of some event to appear, like congestion and high pollution levels; or the possible values of some problems like the travel time for vehicles. Many of the problems inside this category need additional information. In order to provide predictions in a distributed way, information regarding sensors located in the area of interest gathered by a CLU is processed by data mining techniques. With the processing of the collected data, the internal model of the future state of the variable of interest.

In order to make models readable, as well as easy to code and share among CLUs, tree models are used [13]. State of the art decision tree algorithms can be launched both in a local or remote way. Resulting models (tree or set of rules) can be easily shared within all the connected nodes. Some examples of this sort of algorithms can be the well-known C4.5 [14], the ID3 [15] or the Support Vector Machine [16]. This kind of techniques have been successfully applied to a broad range of tasks, from learning to diagnose medical cases [17,18] to school performance prediction [19]. These models are also needed to predict the possible future levels of CO_2_. This fact could be useful for control the restricted areas into the urban use cases of monitoring and reduction of air pollution.

The fourth group of problems is the monitoring group. The monitoring actions taken by the CLU will be oriented to send/receive information about the state of the environment. The collected information will be necessary for a proper decision making to be done by the CLU. Monitoring techniques record the accidents, stopped vehicles on roads, blocked lanes and any other event that could happen in the vehicular flow. It also monitories the slots of all intermodal parking, which is very important to decide correct alternative routes and alternative transports.

## 4. ITS Test Applications and Services

The proposed ITS architecture must be able to prevent and warn about many anomalous situations that can occur in the circulation, as well as to act in consequence. To make this possible, it is necessary to analyse in detail all the possible events, and to select the most effective method or algorithm to solve each use case. The implementation of a set of ITS services running on the proposed architecture has been established in order to validate the correct operation of the whole system.

### 4.1. Use Case Analysis

To perform the tests, a working platform to simulate the necessary information has been created. This platform allows the behaviour analysis of the vehicles before, during and after the incidents. Table 2 shows the different use cases analysed with this platform.

**Figure 4:**
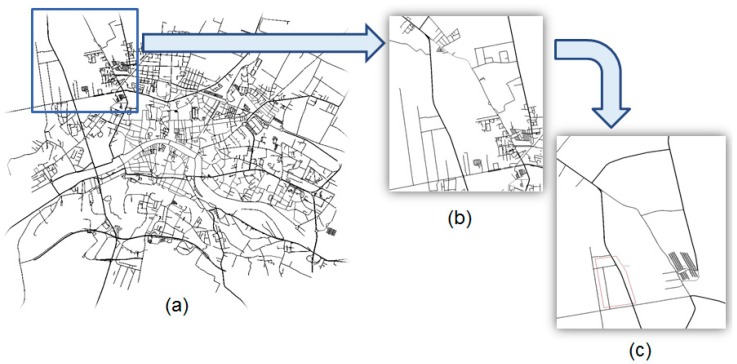
Map in SUMO used for the simulation: (**a**) Pisa city centre. In blue the selected area for the urban use cases simulation; (**b**) Zoom over the selected area, with a 350 slots parking facility; (**c**) Simplified model for the simulations, with the main road connections and the parking facility.

SUMO (Simulation of Urban Mobility) [20], an open source tool for microscopic road traffic simulation, has been used to simulate the behaviour of the vehicles. The solution for urban use cases will be implemented in the city of Pisa, Italy. For this case, Pietrasantina and Aurelia streets have been selected. This selection has been made due to the existence of alternative paths on major roads, as well as an intermodal parking. Figure 4 shows the map used for the urban simulations.

During the simulation, the system monitors the CO_2_ levels of the restricted area. When the system detects that these levels continue to rise, being probable that the maximum levels can be exceeded, the restricted area is closed. Once the restricted area is closed, only authorized vehicles are allowed to access it, while the rest of the vehicles must be re-routed. In addition, the Pietrasantina’s Parking has been defined in a way that the number of free slots is available for simulation purposes. This parking has more than 350 slots. With the aim of simulating intermodal applications, the bus station has been incorporated, carrying drivers from/to the Pisa city centre.

On the other hand, to perform the simulations of the highway use cases, a 10 km section of the A5 highway of Portugal has been used. In this use case, the system continuously receives the events that happen on the road, such as accidents on the road or stopped vehicles. Once an incident is reported to the system, it initiates a process to prevent further damage, and it warns other vehicles as soon as possible. Additionally, for the traffic jam warning use case, the simulator receives the flow of the highway in real time. In this way, when a traffic jam is detected, the system starts the process of alerting nearby vehicles to prevent them entering the jam. In addition, vehicles with the possibility of using an alternative route are informed about the incidence, and provided with the possibility of using an alternative route.

### 4.2. Test Environment

Along this subsection, it will be exposed how the architecture functionality and capabilities were tested. Before the start of the planned field trials, which will be performed in Pisa (Italy) and Lisbon (Portugal), two datasets were created to test the CLU implementation and the operation of the GW. These datasets correspond to the urban and highway scenarios, defining the value of the sensors and the events generated by them for a certain period of time. The objective is to see not only the flow of events generated by the use cases, but rather the communications between CLUs as well:

- Collaborating and communicating with other components of the architecture, with the aim of elaborating environment information in a distributed way.
- Enabling cooperative intelligence, and learning from each situation in order to provide different actions plans depending on the events received from the reality of interest.

**Figure 5:**
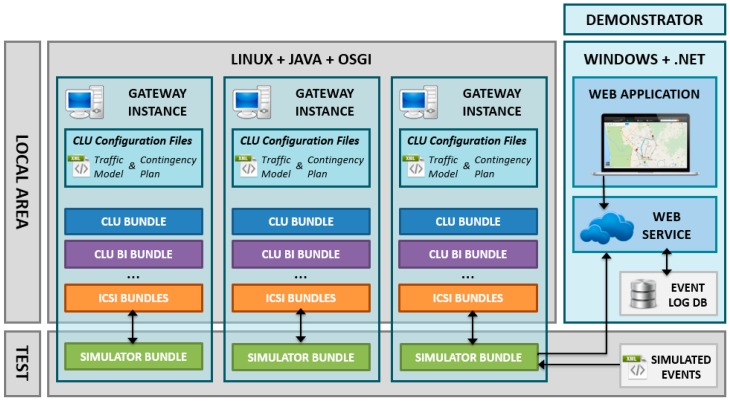
Architecture Test Environment.

Figure 5 shows the global architecture of the designed test environment, which is composed of two main elements: the simulator bundle, an OSGi module integrated in the architecture, which recreates the ingoing events, and the demonstration web service and application, which interacts with the system via the simulation bundle to show real-time values of the sensors.

#### 4.2.1. Simulator Bundle

As can be seen in Figure 5, three GW instances are deployed. These instances are complete SW architecture implementations running in a GNU/Linux machine in three different processes. These processes communicate with each other via event messages. The CLU OSGi set of bundles and the needed configuration files are also included and running on each of the instances, so a complete Local Area is being simulated in the provided test environment.

The CLU Simulator bundle is also included in the instances for testing purposes. It will read the temporally sequenced list of sensor values from the provided use case simulation datasets. Then, using the Event Processing Language (EPL), it will generate the corresponding Data Model to publish the simulated event inside the GW’s Event Manager, just as if it were a real event sent by an external sensor attached to the GW.

The functionality of this bundle is required to adequately test the reception of event messages from the cooperative networks. In the following integration tests (detailed in Section 5) this bundle will be replaced seamlessly with real information coming from the ICSI infrastructure.

#### 4.2.2. Demonstration Web Application

A web application for the real time monitoring of the sensors and actuators has been incorporated to the testing solution. It is possible to monitor in real time the vehicles position, the monitored events (e.g., traffic jam, accidents, etc.) and the messages generated by CLUs on each GW according to the contingency plans.

For vehicles position tracking the positioning information carried by CAM messages generated by On Board Units (OBUs) is used. Each vehicle that is equipped with an OBU can be tracked. This track is possible thanks to the DDP event processor service, which is used to process the CAM messages. These messages are received by the GWs in the RSUs. All of the events generated on a particular GW are also propagated to the other GWs located in the same Local Area, including the one running the web application, in charge of displaying visual information for the monitored area.

**Figure 6:**
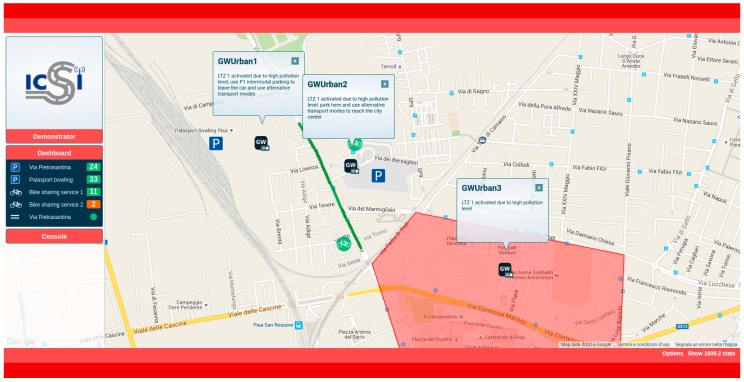
Demonstration Web Application.

To test some of the use cases, the web application offers the capability of generating some of the events that should be triggered by the traffic management centre. In this case, the injected events are also forwarded to the other GWs in the Local Area, where the DDP and the CLU can process them and react properly. Then, the active events in the system can be monitored on the platform, where it is possible to cancel them if necessary (e.g., an accident event can be cancelled by the web application after the accident is clear and the road condition has been restored). With this application (Figure 6), the administrator of the system, or the responsible for carrying out the final deployment, can test the functionality and the correct operation of the system in a simulated environment prior to the physical deployment of the RSUs on the road.

The web application is a Single Page Application developed in C# with a map-based interface based on Google Maps API. The application is a RIA (Rich Internet Application) [21], and follows the MVC architecture. For the presentation layer design, HTML5, CSS3 and client programming languages like JavaScript, JQuery and Ajax have been used. Figure 6 shows a screenshot of the developed demonstration web application.

## 5. Field Experimentation

This section presents the process followed with the aim of testing the architecture functionality using real data coming from predefined ICSI scenarios. As has been mentioned in previous sections, two scenarios have been selected for this experimentation, corresponding to the two field trials scheduled in Pisa (Italy) and Lisbon (Portugal).

For the first test scenario, a location in the access point of the Pisa city centre has been selected. Real data about historical pollution levels in the city has been incorporated to the experimentation. On the other hand, in Lisbon, the system was tested along the A5 highway, and the trials were executed in order to assess the performance of the communications, namely in terms of the IT2S communication. Real data about the vehicles traffic flow in the A5 highway has been incorporated in order to test the CLU in a context as close to the real one as possible.

### 5.1. Urban Scenario in Pisa, Italy

Pisa is a city in Tuscany, central Italy, on the right bank of the mouth of the River Arno on the Tyrrhenian Sea. It is the capital city of the Province of Pisa. In this city, some areas have a controlled access through Restricted Traffic Zones (RTZs) and Low Emission Zones (LEZ). These RTZs are closed to non-residential vehicle traffic.

On the other hand, LEZs are a way to reduce the pressure of non-residential traffic in highly touristic destinations. The objective of LEZs is to control the pollution level in highly congested and populated zones. More in detail, the area selected for this experimentation is located on the access point of the city for most of the traffic from the highly inhabited north costal area, including vehicles exiting at “Pisa Nord” highway exit. Therefore, this road collects both local urban traffic, having source and destination in Pisa area, as well as long distance traffic.

The parking area is close to Pisa main touristic attractions (including the leaning tower), to the hospital facilities, and to several buildings and departments of the local university. Therefore, a mixture of students, commuters and tourists uses this parking area. To foster park-and-ride, the area is served by two bus lines. The first one is dedicated to tourists, and it allows reaching the leaning tower in few minutes. The second one, named LAM Rossa (High Mobility Line RED), goes through the city centre. This second line reaches the main train station after 20 min, and it goes to Pisa airport, which is reached eventually after 25 min from the beginning of the journey.

**Figure 7:**
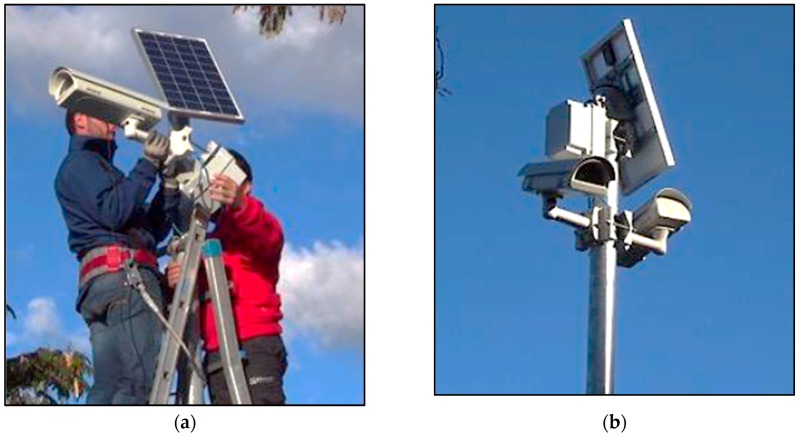
Sensor installation for the urban tests: (**a**) mounting the sensor and the photovoltaic panel on the top of the pole; and (**b**) example of installation of two sensors on a single pole.

In the test location, a portion of the parking lot dedicated to private vehicles is monitored. The plan for the equipment installation included the deployment of 12 sensors on 6 poles for monitoring up to 71 slots. In addition, flow monitoring is performed at the entrance of the city, measuring flows in a location at about 1 km far from the parking lot. Figure 7 illustrates the sensors installation process performed for the trials.

Two GWs have been deployed in the test fields, while a third one has been installed in the TMC in order to monitor the trial and to provide the VPN required exchanging messages among the GWs.

The demonstrator application has been used also in the trials, because of the similarities between the scenarios of the urban trials and the first version of the Demonstration Web Application. In particular, the application has been adapted and extended for the scenario, and it has been deployed in one of the GWs of the urban trial. In this way, the correctness of the event notifications can be monitored.

#### 5.1.1. Long Term Monitoring Results

Several long term tests have been performed in the context of the experimentation. In this section, the obtained results are presented and analysed. Specifically, two separate sets of tests have been performed. The first one related to the flow monitoring. The other one regarding the parking lot occupancy monitoring.

In both field trials, the results cover a two months’ period spanning from 1 November to 31 December 2015. Each of the test sets used different metrics and evaluation methods. In particular, the parking lot tests analysed the occupancy ratio daily rates and trends, while the flow monitoring tests analysed the traffic flow rate on daily and global basis, as well as the average speeds and the cumulative vehicle count curves (so called N-curves).

##### Parking Lot Monitoring Tests

The actual total amount of the slots monitored by the installed sensors and its subdivision among categories is shown in Table 3.

**Figure 8:**
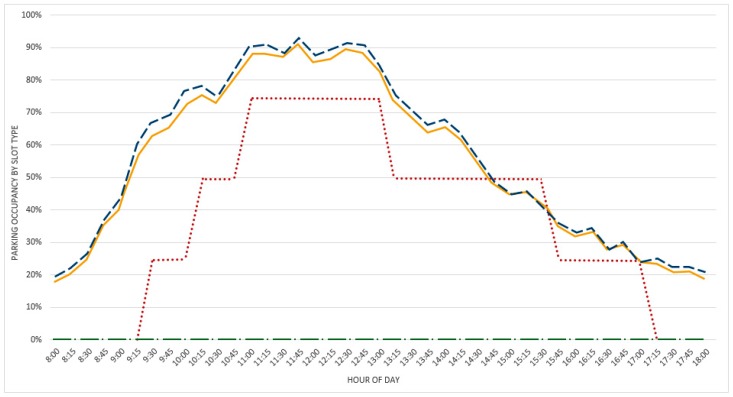
Occupancy status of the parking on 2 November.

The analysis of the results shows the activity of a typical working day. The chosen day for this analysis is 2 November. The monitoring, due to wintertime (i.e., short daylight time) and to the need to save the battery at nighttime, took place from 8:00 a.m. to 6:00 p.m. on each day. In Figure 8, the occupancy status recorded every 15 min is shown.

Obtained results confirm the usage of Via Pietrasantina parking as a typical long-stay swapping parking used also for mobility exchange reasons (e.g., fast bus stop link to centre and train station). In fact, the occupancy ratio reaches its highest values early and quickly in the morning and only decreases slowly in the afternoon to get to its minimum towards the offices closing time.

##### Flow Monitoring Tests

Regarding the flow monitoring tests performed along Via Pietrasantina (the north access way to the city of Pisa), the data acquired covered different aspects: the amount of vehicles, the average speed categories detected, and the aggregation of these data on time slots and daily basis. The first data to report and analyse are the traffic flow rate on a single day.

**Figure 9:**
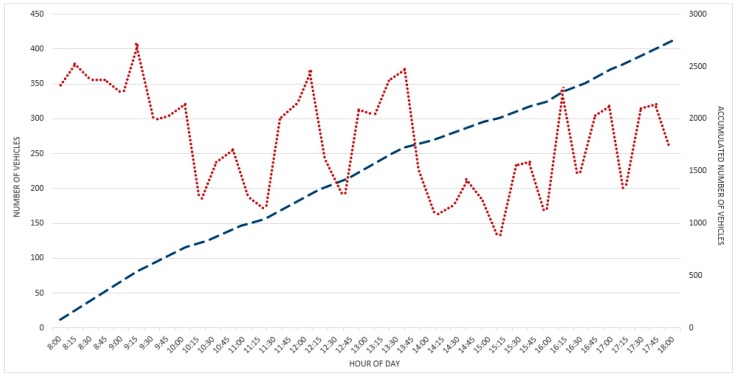
Traffic flow data. Number of vehicles/hour (dotted) and cumulative vehicle count (dashed).

For this purpose, the same day as the parking lot scenario has been chosen: 2 November. In Figure 9, vehicles per hour ratio and N-curve are shown. The data are evaluated on a 15-min basis. As well as the previous tests, the monitoring took place from 8:00 a.m. to 6:00 p.m. on each day.

As it was expected, the flow ratios in a normal working day have the highest values in the early morning hours to quickly decrease at around 10:00 a.m. Then, these values increase again during lunchtime (i.e., that is also mostly closing school time). After that, a rapid decrease happens around 2:00 p.m., increasing slightly again after 4:00 p.m. for the closing office time, which is more distributed in time (e.g., spanning from 4:00 to 8:00 p.m.). Another interesting datum is the total amount of vehicles. In that day, the amount was 2749, in range of the typical working day data.

**Figure 10:**
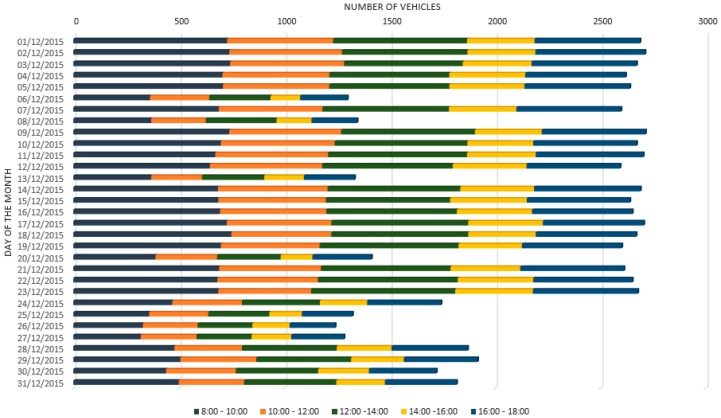
Traffic flow per day. Accumulated number of vehicles/hour in December.

Besides that, a brief analysis of the aggregated results brings to some remarks: differently from the parking lot tests, the traffic flow presented in Figure 10 shows the bigger decrease only on Sundays and holidays. On the other hand, on Saturdays, a traffic flow comparable to other working days can be seen. Sundays and holidays (i.e., 8 December and, 25 and 26 December, respectively) see the average traffic flow ratio to fall more than 50%. Moreover, similar to the parking lot scenario, during Christmas holidays (i.e., after 23 December) there is a decrease around 30% of the traffic flow.

#### 5.1.2. Urban Use Cases Test Results

In this context, and having the collected data about the traffic flow and the parking availability, the proposed urban test scenario includes the implementation of the following use cases:

- Alternative transport services;
- Monitoring and reduction of air pollution;
- Alternative paths signalling/route guidance; and
- Cooperative parking slots monitoring.

The system constantly monitors the pollution of the roads in the RTZ and LEZs of Pisa. As has been explained before, when it predicts that the level of pollution can exceed the threshold, it suggests leaving the car in the parking area, continuing the trip using alternative transport services. In addition, the system estimates in real-time the number of free slots in the parking lot. In this way, it can recommend the most appropriate parking lot to leave the vehicle. This fact highlights the opportunity to provide intermodal transport solutions. Table 4 shows the tasks performed by the different components of the architecture in order to accomplish the requirements of the test scenario.

**Figure 11:**
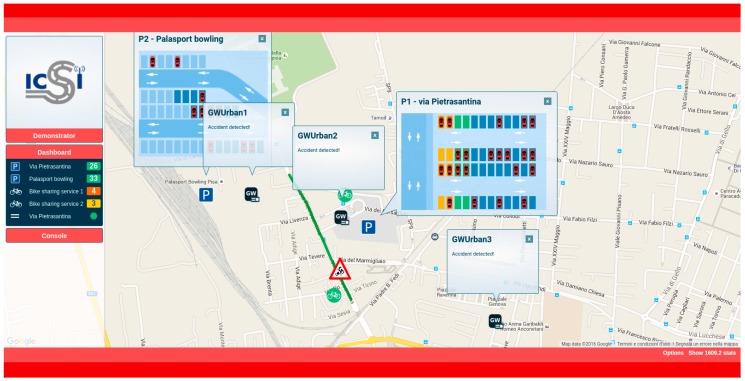
Demonstration Web Application in the urban test scenario.

The main goal of the experimentation is to show that the system, by the aggregation of distributed sensors data and the implementation of collaborative intelligence, can provide relevant information related to the analysed use cases to the users, and therefore improve their decision making while using their vehicles. The verification of some of these use cases have been reproduced in the laboratory due to the need of producing events related to current pollution levels. Figure 11 shows a snapshot of the demonstrator application GUI for the urban test scenario.

Three different GWs are available in this scenario. GW1 and GW2, receiving the status of parking slots, and GW3 receiving data about actual pollution. These GWs are configured to listen for coming events from their own attached sensors. All of the GWs calculate the *Parking Occupancy* and predict *Pollution Levels*. For this urban scenario, two data sources have been used. The first one is the number of free parking slots. The other one is the pollution level in the LEZ. It is interesting to point that historical data about pollution in the city of Pisa is used here. These data have been provided by INTECS (an Italian ICT company focused in the design of SW/HW electronic components, and partner of the ICSI project).

### 5.2. Highway Scenario in Lisbon, Portugal

**Figure 12:**
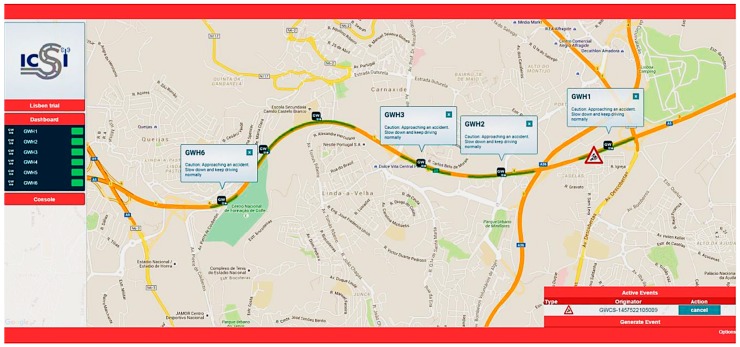
Demonstration web application with the RSU locations for the highway scenario.

**Figure 13:**
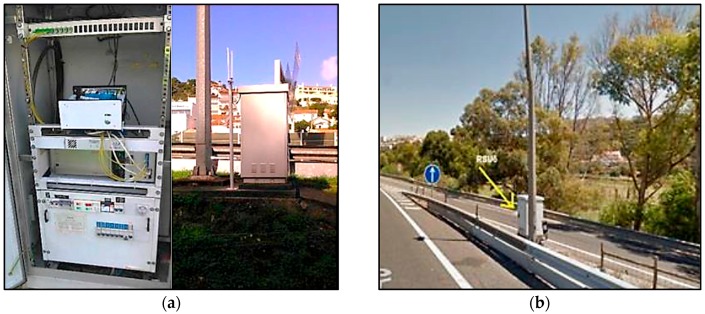
Field equipment installation for the highway tests: (**a**) RSU inside the cabinet and 5.9 GHz and GPS antennas outside; and (**b**) RSU installed in the A5 highway, at Kilometre 7.4.

The A5 highway of Lisbon is a 25 km (16 miles) long motorway that connects the capital city of Portugal to Cascais. The first section of this infrastructure was opened in 1944, becoming the first motorway in Portugal and one of the firsts in the world. Nowadays, it is the most travelled motorway of the country and one of the most congestion prone ones. Six GWs were installed (location marked in Figure 12) and mounted on road side cabinets (photos of the installed equipment in Figure 13).

The RSUs were interconnected by TCP-IP network, and together with the GWs made possible the implementation of the platform on the field trial location. The ITS G5 stations have been configured as RSU and mounted inside the road side equipment cabinets along the A5. These cabinets already have power and TCP/IP communication, being only necessary to install the radio and GPS antenna. The RSU platform is composed of the SoC and the ETSI-G5 Radio Board. In addition, two antennas were used, one omnidirectional of 5.9 GHz with 12 dBi gain, and a GPS external antenna. The communication with the GW is made through optical fibre.

The ITS G5 stations configured as OBU were installed in BRISA Innovation vehicles models (BRISA is a Portugal-based international transportation company, and partner of the ICSI project). Each of these vehicles is equipped with a 5.9 GHz antenna placed in the roof, as well as an external GPS antenna. This antenna is in charge of synchronizing communications and providing GPS data. On the other hand, the OBU is powered by the vehicle battery. The system has a Human Machine Interface (HMI). This HMI is a mobile application that runs in a smartphone connected to the OBU.

**Figure 14:**
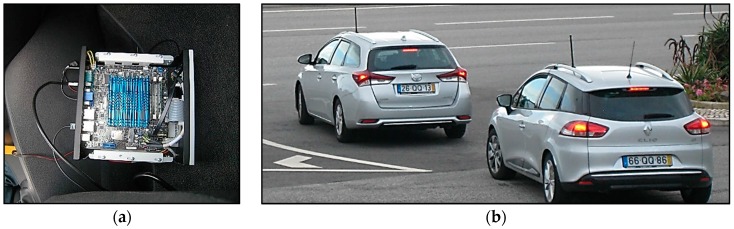
On-board equipment installation for the highway tests: (**a**) OBU installed in the vehicle; and (**b**) two vehicles equipped with the antennas ready for the tests.

This application allows the driver to see and report Road Hazard Warnings (RHWs) sent by/to the vehicle network on the different tested use cases. The smartphone was placed inside the vehicle, near the dashboard, making easily reachable to the driver. Figure 14 shows the installed equipment.

#### 5.2.1. Platform Performance Evaluation

As mention before, for the highway field trials, six road side units were installed on cabinets in different positions along the A5 highway. On the other hand, RSUs are positioned at different heights, suffering different interferences due to nearby objects and road geometry (curve radius, inclination, declination, etc.). One of the parameters evaluated on this test was the RSUs coverage area.

**Figure 15:**
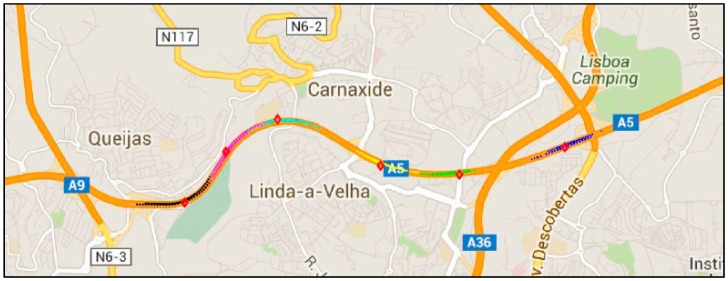
Coverage area of the RSUs installed for the highway field trial.

Figure 15 shows the RSUs coverage area on the A5 highway. Every dot on the chart represents the GPS location of the OBUs when a message was received by the RSU. It is possible to see that the area of the highway between Queijas and Lisboa Camping is not totally covered by the installed RSUs. In addition, we can observe the differences in coverage areas length, and the overlaps that exist in some of these coverage areas. This overlap can lead to double notifications of the events (produced by the overlapped RSUs) that should be managed by the CLUs.

#### 5.2.2. Highway Use Cases Test Results

In this context, and with the installed communication devices ready for the trials, the proposed highway test scenario includes the implementation of the following use cases:

- Monitoring of anomaly in traffic flows (congestion);
- Accident warning; and
- Road works warning.

**Figure 16:**
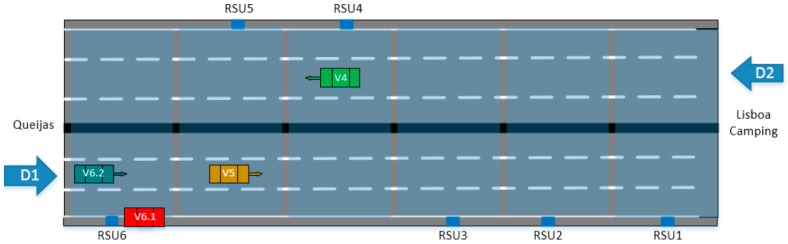
Highway scenario, traffic jam use case, A5 Portugal.

The proposed ITS distributed architecture provides in-route traveller information about traffic and road conditions according to both static and dynamic rules. In this way, drivers who are approaching a traffic jam can take some precaution measures, like reducing the speed in advance. The system tries to avoid these problems, warning the drivers about the traffic jam, even before it could be noticed by the driver. Thus, drivers are informed in time, and they can react smoothly and safe. Figure 16 shows a diagram of the highway scenario with the location of the vehicles approaching a congested area. The deployed RSUs on the road are also included.

A total of six different RSUs are used in this scenario, all of them with the same configuration. Thus, each RSU’s GW is configured to get *Abnormal Traffic* events (e.g., accident or roads work warning) from the next GW on the road. Additionally, each GW also gets *Vehicle Counter* events. These events come from the GW’s attached sensors, and they are delivered to the CLU in order to detect congestion using the implemented artificial intelligence. In line with this, if congestion is detected, a *Congestion Level* event is launched. Each GW is listening for *Congestion Level* events from itself and from the next GW on the road in order to act with foresight and warn the drivers about expected traffic jumps.

The traffic jam warning is implemented on the platform based on the above described traffic model. Around 1000 position events with different station ID are necessary to trigger the traffic jam warning (CAM messages) for 30 min. Based on the vehicles used on the ICSI trial, it was not possible to test this use case. To solve this problem, we have changed one of the OBU software packages in order to generate on its own these 1000 position events. This OBU has been installed on a vehicle that was stopped near RSU6, outside the highway. Every vehicle that approaches this zone will then receive the traffic jam warning. The vehicle (V6.1) that will trigger the dummy position events is stopped near to the RSU6. Every vehicle (as for example, V6.2) that is approaching this location receives the traffic jam warning. In addition, the TMC will also receive the warning.

**Figure 17:**
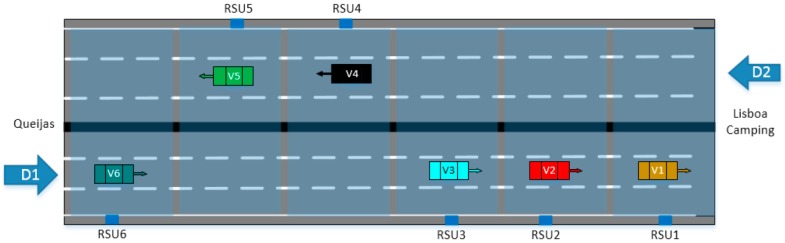
Highway scenario, accident warning use case, A5 Portugal.

Furthermore, accidents occur every day on highways. Some of them could be avoided if warnings could be sent to alert road users about the proximity of the accident zone, allowing them to take preventive measures. The TMC informs the road users through the variable message panels, but usually there is no panel installed on the accident location, or near. In line with this, the proposed system can help to solve this problem, sending an accident warning message to the road users that are approaching the accident zone, in order to take safety precautions. Figure 17 shows the experimentation performed in the highway scenario to test the accident use case.

The TMC detects the accident and informs the road users through the developed platform, sending the accident warning message to the RSUs. The road users will be informed by an accident warning message at their HMI devices when they are approaching the accident zone covered by the platform. In this way, precautions can be taken to avoid other accidents.

To validate this use case, the TMC has been used to trigger a simulated accident warning message through the platform. This message indicated an accident of V1 in direction D1. Based on the available data, the implemented CLU is able to alert the drivers and/or the emergency services. Vehicles approaching the accident zone (V2, V3 and V6) will be informed, receiving the warning message from the RSUs installed on the road. Vehicles V4 and V5 will not be informed as they are driving in opposite direction.

The produced messages have been also successfully received at the GUI Web Platform and the HMI, included as a mobile application inside the vehicles, informing that it is recommended to take exit to avoid traffic congestion or alerting about an accident with foresight.

In this way, the following tasks (Table 5) have been successfully validated in the test scenario.

**Figure 18:**
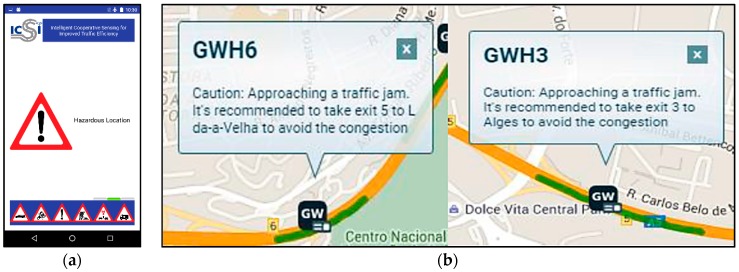
Alert messages received by the user: (**a**) HMI installed in the vehicle; and (**b**) fragment of the demonstrator web application.

Figure 18 shows a snapshot of the demonstrator application GUI and the HMI for the highway test scenario.

## 6. Conclusions

This work, based on the ICSI European project, exposes a new paradigm in the ITS domain, moving the system intelligence from the control centre to the peripheral devices. The presented architecture uses a set of gateways, installed in the road infrastructure to process and gather all the sensors data independently and in a distributed way, without the need to contact a central subsystem.

The presented architecture, with the participation of the sensors networks on the road, the data distribution platform, the collaborative learning units and, finally, the ITS applications, encompasses the entire process of capture and management of available road data, enabling the generation of services to promote transportation efficiency and performance, among others.

This paper provides a description of the overall system architecture explaining the role of the different elements of the system and the way the intelligence and the sensed-data are provided. Furthermore, a more specific view of the Collaborative Learning Unit, the component responsible for providing intelligence to the system, is presented. Besides that, the developed demonstration environment has been presented.

This environment counts with a web application that is able to display the complete operation of the CLUs with real time monitoring of the sensors, actuators, vehicles positions, and the messages generated by CLUs on each GW.

Finally, the development of a set of field trials performed in Italy and Portugal under the collaboration in the ICSI European project is exposed, fulfilling the main goal of the project. This main goal is the deployment of cooperative systems, based on V2X communication technologies, with the aim of enabling safer and more efficient mobility.

The proposed system is able to demonstrate the feasibility of a flexible and innovative platform from the architectural point of view that has been specialized for the ITS context, but generally applicable in other contexts and compatible with the Internet of Things paradigm, allowing considerable flexibility and scalability of the system.

## Figures and Tables

**Table 1:** Different Problematic being faced by CLU.

Category	Problems Faced	Techniques
Routing	- Alternative routes - Alternative transport - Route guidance	- Genetic Algorithms - Fuzzy Decision Support Systems - Probabilistic Models
Regulation	- Ramp-metering algorithms	- Fuzzy Control
	- Access to pollution-monitored area	- Hybrid Models
		- Particle Swarm Optimization
Prediction	- Level of congestion - Travel time for vehicles - Pollution	- Probabilistic Models - Bio-inspired optimization - Time-series prediction
Monitoring	- State of traffic - Free parking slots - Incidents	- Particle filters - I2V Communications

**Table 2:** Use cases analysed to test the proposed architecture.

Identifier	Type	Description
UC1	Urban	Monitoring and reduction of air pollution
UC2	Urban	Cooperative parking lots monitoring
UC3	Highway	Alternative paths signalling
UC4	Highway	Monitoring of anomaly in traffic flows
UC5	Highway	Traffic jam warning

**Table 3:** Actual monitored slots composition.

Total Slots	Regular Slots	Disabled People Slots	E-Car Slots
71 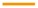	66 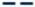	4 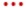	1 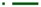

**Table 4:** Urban scenario related tasks.

Task	Test Equipment			
	GW	Sensor	GUI	CLU
Traffic flow monitoring in the selected area		√		
Parking space vacancy monitoring in via *Pietrasantina* and *Palasport* parking areas		√		
Air pollution level monitoring in city centre		√		
Data management and event publishing	√			
Event processing, alert messages generation				√
Evolution of congestion and pollution levels prediction for the next period of time				√
Suggestion of new parking area target				√
Display the availability of both parking areas			√	
Display the LTZ area in the map			√	
Suggestion of alternative transport modes			√	

**Table 5:** Highway scenario related tasks.

Task	Test Equipment				
	GUI	RSU	OBU	CLU	DDP
Trigger the event (provided by TMC)	√				
Trigger the event (provided by the vehicle)			√		
Receive the event over-the-air and send it to the GW		√			
Receive the event, process it and generate the congestion alert message. Send it to the RSUs				√	√
Receive the message and send it over-the-air to the OBUs		√			
Message received in vehicles (in the same direction)			√		
Message NOT received in vehicles in opposite direction			√		
Receive and display the congestion and alert messages in the map	√				
