# Correction: Moreno, A., et al. Design of a Cooperative ITS Architecture Based on Distributed RSUs. † *Sensors* 2016, *16*, 1147

**DOI:** 10.3390/s17061301

**Published:** 2017-06-06

**Authors:** Asier Moreno, Eneko Osaba, Enrique Onieva, Asier Perallos, Giovanni Iovino, Pablo Fernández

**Affiliations:** 1 DeustoTech—Fundación Deusto, Avda. Universidades, 24, 48007 Bilbao, Spain; 2 Faculty of Engineering, University of Deusto, Avda. Universidades, 24, 48007 Bilbao, Spain; asier.moreno@deusto.es (A.M.); e.osaba@deusto.es (E.O.); enrique.onieva@deusto.es (E.O.); pablo.fernandez@deusto.es (P.F.); 3 INTECS SpA, Via Umberto Forti 5, 56121 Pisa, Italy; giovanni.iovino@intecs.it

The authors wish to make the following corrections to this paper:

(1) The work described in this paper encompasses part of the European Intelligent Cooperative Sensing for Improved (ICSI) traffic efficiency project. The authors, as partners of the project, want to clarify that its main contribution, which is the central part of the paper, is the cooperative ITS architecture design and implementation. Specifically, the implementation of the Collaborative Learning Unit (CLU). Accordingly, the authors have decided to reduce and modify Section 5, that should be removed and replaced with the following:

## 5. Field Experimentation

This section presents a summary of the results and the process followed with the aim of testing the architecture functionality using real data coming from predefined ICSI scenarios. As has been mentioned in previous sections, two scenarios have been selected for this experimentation, corresponding to the two field trials scheduled in Pisa (Italy) and Lisbon (Portugal).

For the first test scenario, a location in the access point of the Pisa city centre has been selected. Real data about historical pollution levels in the city has been incorporated to the experimentation. On the other hand, in Lisbon, the system was tested along the A5 highway, and the trials were executed in order to assess the performance of the communications, namely in terms of the IT2S communication. Real data about the vehicles traffic flow in the A5 highway has been incorporated in order to test the CLU in a context as close to the real one as possible.

The main goal of the experimentation is to show that the system, by the aggregation of distributed sensors data and the implementation of collaborative intelligence, can provide relevant information related to the analysed use cases to the users, and therefore improve their decision making while using their vehicles.

### 5.1. Urban Scenario in Pisa, Italy

Pisa is a city in Tuscany, central Italy, on the right bank of the mouth of the River Arno on the Tyrrhenian Sea. It is the capital city of the Province of Pisa. In this city, some areas have a controlled access through Restricted Traffic Zones (RTZs) and Low Emission Zones (LEZ). These LEZs are a way to reduce the pressure of non-residential traffic in highly touristic destinations. The objective of LEZs is to control the pollution level in highly congested and populated zones. In this context, and having the collected data about the traffic flow and the parking availability, the proposed urban test scenario includes the implementation of the following use cases:

- Alternative transport services;
- Monitoring and reduction of air pollution;
- Alternative paths signalling/route guidance; and
- Cooperative parking slots monitoring.

The system constantly monitors the pollution of the roads in the RTZ and LEZs of Pisa. As has been explained before, when it predicts that the level of pollution can exceed the threshold, it suggests leaving the car in the parking area, continuing the trip using alternative transport services. In addition, the system estimates in real-time the number of free slots in the parking lot. In this way, it can recommend the most appropriate parking lot to leave the vehicle. This fact highlights the opportunity to provide intermodal transport solutions. Table 3 shows the tasks performed by the different components of the architecture in order to accomplish the requirements of the test scenario.

The verification of some of these use cases have been reproduced in the laboratory due to the need of producing events related to current pollution levels.

### 5.2. Highway Scenario in Lisbon, Portugal

The A5 highway of Lisbon is a 25 km (16 miles) long motorway that connects the capital city of Portugal to Cascais. The first section of this infrastructure was opened in 1944, becoming the first motorway in Portugal and one of the firsts in the world. Nowadays, it is the most travelled motorway of the country and one of the most congestion prone ones. Six GWs were installed on the motorway road side cabinets. The RSUs were interconnected, and together with the GWs made possible the implementation of the platform on the field trial location.

Field trials were performed by partners of the ICSI project (IT, BRISA and INTECS). The following summarizes the tested use cases, not being the purpose of this work the detailed description of those trials, which will be subject of forthcoming works. In this context, the proposed highway test scenario includes the implementation of the following use cases:

- Monitoring of anomaly in traffic flows (congestion);
- Accident warning; and
- Road works warning.

The proposed ITS distributed architecture provides in-route traveller information about traffic and road conditions according to both static and dynamic rules. In this way, drivers who are approaching a traffic jam can take some precaution measures, like reducing the speed in advance.

Each RSU’s GW is configured to get *Abnormal Traffic* events (e.g., accident or roads work warning) from the next GW on the road. Additionally, each GW also gets *Vehicle Counter* events. These events come from the GW’s attached sensors, and they are delivered to the CLU in order to detect congestion using the implemented artificial intelligence. In line with this, if congestion is detected, a *Congestion Level* event is launched. Each GW is listening for *Congestion Level* events from itself and from the next GW on the road in order to act with foresight and warn the drivers about expected traffic jumps.

The produced messages have been also successfully received at the GUI Web Platform and the HMI, included as a mobile application inside the vehicles, informing that it is recommended to take exit to avoid traffic congestion or alerting about an accident with foresight. Figure 7 shows a snapshot of the demonstrator application GUI for the highway test scenario.

(2) The title, according to the changes made to the paper, must be replaced with:
  “Design of a Cooperative ITS Architecture Based on Distributed RSUs.”
(3) The abstract should be modified as follows:
  The sentence:
  “Finally, functional and operational results observed through the experimentation are described.”
  should be removed and replaced with:
  “Finally, the process followed with the aim of testing the architecture functionality is described.”
(4) Author contributions should be replaced with the following:
  **Author Contributions:** Asier Moreno and Eneko Osaba wrote the paper. Asier Perallos, Asier Moreno and Giovanni Iovino designed and conceived the architecture. Enrique Onieva and Pablo Fernandez conceived and designed the experiments related to CLU testing. Giovanni Iovino in collaboration with the partners of the ICSI project (CNR, CNIT, IT and Brisa) performed the experiments. All authors supervised the paper and provided substantive comments.

The authors apologize for any inconvenience caused to readers.

## Figures and Tables

**Figure 7:**
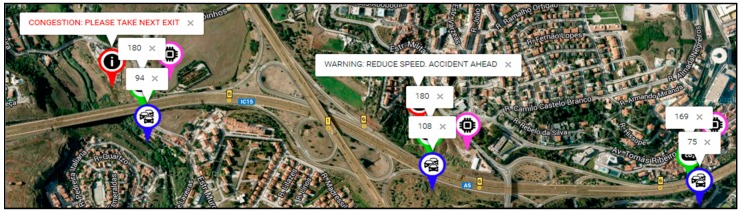
Alert messages received by the user: fragment of the demonstrator web application.

**Table 3:** Urban scenario related tasks.

Task	Test Equipment			
	GW	Sensor	GUI	CLU
Traffic flow monitoring in the selected area		√		
Parking space vacancy monitoring in via *Pietrasantina* and *Palasport* parking areas		√		
Air pollution level monitoring in city centre		√		
Data management and event publishing	√			
Event processing, alert messages generation				√
Evolution of congestion and pollution levels prediction for the next period of time				√
Suggestion of new parking area target				√
Display the availability of both parking areas			√	
Display the LTZ area in the map			√	
Suggestion of alternative transport modes			√	

